# Directed evolution of *Zymomonas mobilis* sugar facilitator Glf to overcome glucose inhibition

**DOI:** 10.1093/jimb/kuab066

**Published:** 2021-09-16

**Authors:** Gavin Kurgan, Moses Onyeabor, Steven C Holland, Eric Taylor, Aidan Schneider, Logan Kurgan, Tommy Billings, Xuan Wang

**Affiliations:** School of Life Sciences, Arizona State University, Tempe, AZ 85287, USA; School of Life Sciences, Arizona State University, Tempe, AZ 85287, USA; School of Life Sciences, Arizona State University, Tempe, AZ 85287, USA; School of Life Sciences, Arizona State University, Tempe, AZ 85287, USA; School of Life Sciences, Arizona State University, Tempe, AZ 85287, USA; School of Life Sciences, Arizona State University, Tempe, AZ 85287, USA; School of Life Sciences, Arizona State University, Tempe, AZ 85287, USA; School of Life Sciences, Arizona State University, Tempe, AZ 85287, USA

**Keywords:** Lignocellulose, Directed evolution, Sugar facilitator Glf

## Abstract

Cellular import of D-xylose, the second most abundant sugar in typical lignocellulosic biomass, has been evidenced to be an energy-depriving process in bacterial biocatalysts. The sugar facilitator of *Zymomonas mobilis*, Glf, is capable of importing xylose at high rates without extra energy input, but is inhibited by D-glucose (the primary biomass sugar), potentially limiting the utility of this transporter for fermentation of sugar mixtures derived from lignocellulose. In this work we developed an *Escherichia coli* platform strain deficient in glucose and xylose transport to facilitate directed evolution of Glf to overcome glucose inhibition. Using this platform, we isolated nine Glf variants created by both random and site-saturation mutagenesis with increased xylose utilization rates ranging from 4.8-fold to 13-fold relative to wild-type Glf when fermenting 100 g l^–1^ glucose–xylose mixtures. Diverse point mutations such as A165M and L445I were discovered leading to released glucose inhibition. Most of these mutations likely alter sugar coordinating pocket for the 6-hydroxymethyl group of D-glucose. These discovered glucose-resistant Glf variants can be potentially used as energy-conservative alternatives to the native sugar transport systems of bacterial biocatalysts for fermentation of lignocellulose-derived sugars.

## Introduction

Bioconversion of lignocellulose represents a renewable production route for many petroleum-derived chemicals that does not compete with food crop production. However, the utilization of lignocellulose by microbes is limited by both heterogeneous sugar composition and the toxicity of lignocellulosic hydrolysates following thermochemical pretreatments (Mills et al., 2009; Nieves et al., 2015). For typical agricultural wastes such as corn stover and sugarcane bagasse, their lignocellulosic sugars mainly consist of D-glucose and D-xylose, and coutilization of these sugars is challenging, especially for xylose, due to carbon catabolite repression (CCR), a global transcriptional control for sugar catabolism (Deutscher, 2008; Gorke & Stulke, 2008). CCR results in sequential and suboptimal utilization of the secondary sugars such as xylose and arabinose, which ultimately decrease production metrics. We recently discovered that specific mutations in transcriptional regulator XylR (P363S and R121C; denoted as XylR*) releases CCR in *Escherichia coli* and enables coutilization of lignocellulosic sugar mixtures (Martinez et al., 2019; Sievert et al., 2017). However, besides native transcriptional regulatory mechanisms restricting xylose fermentation, there are at least two intrinsic biochemical mechanisms limiting xylose uptake and thus efficient bioconversion.

First, bacterial pentose uptake in general is not an energy efficient process. Although high concentrations of extracellular xylose are often present during fermentation using lignocellulosic sugar mixtures, most bacterial biocatalysts such as *E. coli* still use active xylose transporters, which results in unnecessary energy consumption (either ATP or proton gradients). In *E. coli*, xylose uptake is achieved primarily by XylFGH, an ATP-binding cassette transporter that expends one ATP per molecule of D-xylose imported (Gonzalez et al., 2017; Hasona et al., 2004; Khankal et al., 2008). Alternative xylose transporters driven by a proton gradient such as XylE and AraE are also employed (Hasona et al., 2004; Henderson, 1990). Following import, xylose is isomerized and phosphorylated with ATP before proceeding into the pentose phosphate pathway. Utilization of these native transport systems has been shown to approximately double the amount of ATP spent on substrate uptake relative to other more efficient sugar uptake systems, such as the phosphoenolpyruvate (PEP): carbohydrate phosphotransferase system (PTS) for glucose uptake and phosphorylation (Gonzalez et al., 2017; Gosset, 2005). For instance, one glucose converted to pyruvate using the PTS system will theoretically yield two net ATPs, whereas one xylose converted to pyruvate using XylFGH on average will yield only 0.67 net ATP (Hasona et al., 2004; Utrilla et al., 2012). Since ATP is limited under anaerobic fermentation conditions (Gonzalez et al., 2017; Hasona et al., 2004), a uniporter for xylose uptake using xylose concentration gradients to drive transport will be desired for lignocellulosic bioconversion.

Second, glucose inhibits the activities of transporters for secondary sugars. For example, the substrate spectra of a broad range of eukaryotic sugar uniporters have been characterized (Young et al., 2011), however many of these transporters have low transport kinetics for xylose and their transport activities are usually inhibited by glucose (Chen et al., 2015; Young et al., 2011, 2014). Glucose inhibition results in sequential sugar utilization and decreased production metrics for multiple biocatalysts in glucose–xylose mixtures, and thus has been the target of protein engineering (Jojima et al.,, 2010; Shin et al., 2015; Young et al., 2012). Similarly, bacterial xylose transporters such as XylE are also inhibited by glucose likely due to structural similarity between glucose and xylose (Sun et al., 2012). An energy efficient xylose transporter mechanistically resistant to glucose inhibition will be useful for fermentations using lignocellulosic sugar mixtures.

Dr. Ingram and his colleagues functionally expressed *Zymomonas mobilis* glucose facilitator gene (*glf*) in *E. coli* (Snoep et al., 1994) and characterized its transport mechanism (Parker et al., 1995). Glf is the only known bacterial glucose facilitator functioning as sugar uniporter to catalyze facilitated diffusion of monosaccharides, and it belongs to the major facilitator superfamily (MFS). MFS proteins usually contain 12 transmembrane spans (TMs) that potentially recognize sugars at the C-terminal domains and facilitate translocation by a rocking mechanism in the N-terminal domains (Chen et al., 2015; Pao et al., 1998). Due to its passive transport mechanism, Glf seems to be an energy efficient alternative to other energy costly uptake system (Tang et al., 2013).

Glf has been shown to have xylose transport activities and mutations (A18T and V275F) were identified to enhance pentose import, suggesting that transport kinetic properties for xylose can be enhanced through mutagenesis (Dunn & Rao, 2015). However, like other MFS sugar uniporters, it is also inhibited by glucose (Chen et al., 2009; Ren et al., 2009). In this work, we identified Glf variants resistant to glucose inhibition through directed evolution using a CCR-free platform *E. coli* that is engineered to be deficient in glucose and xylose uptake. The discovered mutations that confer resistance phenotype provide valuable insights into the structure and function of Glf as a bacterial MFS sugar facilitator.

## Materials and Methods

### Strains, Plasmids, and Media

All strains and plasmids used in this work are listed in Table 1. All genetic manipulations were performed using Luria Broth (10 g l^–1^ Difco tryptone, 5 g l^–1^ yeast extract, and 5 g l^–1^ NaCl). Cells in liquid culture were grown at 30°C, 37°C, or 39°C with rotation at 180 rpm. During genetic manipulations, 50 g l^–1^ arabinose was used to induce recombination and ampicillin (100 mg l^–1^), chloramphenicol (50 mg l^–1^), or kanamycin (50 mg l^–1^) were supplemented as needed.

**Table 1:** Strains and Plasmids Used in This Study

	Relevant characteristics	Reference
Strains		
Top 10F′	F´*lacIq*, Tn10(TetR) *mcrA* Δ(*mrr-hsdRMS-mcrBC*) Φ80*lacZ*ΔM15 Δ*lacX74 recA1 araD139* Δ(*ara leu*) 7697 *galU galK rpsL* (StrR) *endA1 nupG*	Pharmacia
ATCC 9637	Wild-type *Escherichia coli* W	ATCC
WTxyl4	ATCC 9637 xylR::xylR* Δ*ptsI* Δ*ptsG* Δ*galP*	Flores et al. (2019)
EG21	WTxyl4 *xylFGH*::FRT	This study
EG23	WTxyl4 *xylFGH*::FRT *xylE*::FRT	This study
EG25	WTxyl4 *xylFGH*::FRT *xylE*::FRT *araE*::FRT	This study
EG27	WTxyl4 *xylFGH*::FRT *xylE*::FRT *araE*::FRT *araFGH*::FRT	This study
EG29	WTxyl4 *xylFGH*::FRT *xylE*::FRT *araE*::FRT *araFGH*::FRT *gatC*::FRT	This study
EG51A	EG29 *glk*::*kan*	This study
Plasmids		
pKD46	bla, γ β exo (Red recombinase)	Datsenko and Wanner (2000)
pCP20	*bla, cat*, FLP+, ts-rep, cI857λts	Datsenko and Wanner (2000)
pKD4	*bla*, FRT-kan-FRT	Datsenko and Wanner (2000)
pTrc99A	P*trc bla oriR rrnB lacI^q^*	Amann et al. (1988)
pGlf_wt_	*glf* from *Zymomonas mobilis* ZM4 cloned into pTrc99A	This study
pGK1	Isolated plasmid encoding N316S K458R Glf variant	This study
pGK2	Isolated plasmid encoding I170T G313S A344S Glf variant	This study
pGK3	Isolated plasmid encoding L445I Glf variant	This study
pGK4	Isolated plasmid encoding G35V L104S S392C N240S Glf variant	This study
pGK7	Isolated plasmid encoding A18T L116F K149R V218M K357E F374S G422S Glf variant	This study
pGK8	Isolated plasmid encoding T11M N316D K458I Glf variant	This study
pGK14	Isolated plasmid encoding T70I V275D Glf variant	This study
pSM1	Isolated plasmid encoding V162G Glf variant	This study
pSM3	Isolated plasmid encoding A165M Glf variant (pGlf_A165M_)	This study
pGlf_A18T_	Constructed plasmid encoding A18T Glf variant	This study
pGlf_V275F_	Constructed plasmid encoding V275F Glf variant	This study
pGlf_A165M K458I_	Constructed plasmid encoding A165M K458I Glf variant	This study
pGlf_A165M K458I N316D_	Constructed plasmid encoding A165M K458I N316D Glf variant	This study
pGlf_A165M L445I_	Constructed plasmid encoding A165M L445I Glf variant	This study

### Methods for Gene Inactivation

Gene inactivation was performed using one-step inactivation as previously described (Datsenko & Wanner, 2000). Linear DNAs for integration were amplified from pKD4 for primary integration (FRT-*kan*-FRT). All linear DNA fragments were flanked by 50 bp of homology to the region of interest. The *kan* cassette was removed by FLP-promoted recombination using pCP20. All genetic manipulations were verified using colony PCR. All primers used for gene inactivation are listed in Table S1.

### Plasmid Library Construction and Selection Process

To create Glf variants, we took a two-pronged approach employing both random and site-saturation mutagenesis. Circular polymerase extension cloning (CPEC) (Quan & Tian, 2011) was used to clone wild-type *glf* gene and create Glf plasmid library. Primers used for cloning and library construction are listed in Table S1. Wild-type *glf* gene was amplified from *Z. mobilis* CP4 with 20 bp native flanking sequences to the coding region and cloned into pTrc99A backbone to generate pGlf_wt_. For random mutagenesis, the *glf* gene with random mutations with ∼20 bp homology overhangs was assembled with a linearized pTrc99A backbone. Error-prone PCR (ePCR) was used to generate *glf* variants using the GeneMorph II random mutagenesis kit (Agilent) according to the manufacturer instructions. Approximately 12 000 plasmid variants were created in total through multiple rounds of ePCR and CPEC (GK plasmid library).

For site-saturation mutagenesis, we used PCR to generate NNK variants using degenerate primers at the codons that correspond to A162, A165, F374, and G379 in Glf and the resulting plasmid sublibraries (more than 200 independent clones for each site) were constructed as previously described (Reetz & Carballeira, 2007). All sublibraries obtained using site-saturation mutagenesis were pooled into a single tube at equimolar amounts to create the final library (SM plasmid library).

Each library was transformed into EG51A to achieve at least fivefold coverage for resulting transformants and cells were immediately transferred from plates into fermentation medium supplemented with a mixture of 66 g l^–1^ glucose and 34 g l^–1^ xylose. By transferring these fermentation cultures into a new vessel with fresh medium every 24 h for 3 or 4 times, cells with Glf variants that grew more rapidly in the presence of glucose were enriched and these Glf variants may have increased resistance to glucose inhibition. Individual plasmids were then isolated from resulting cultures and Sanger sequencing was used to confirm unique Glf variants. All unique plasmids were retransformed into EG51A to confirm their positive phenotype.

### Construction of Plasmids Encoding Site-Directed *glf* Mutations

Glf plasmids with directed mutation (A165M K458I, A165M K458I N316D, and A165M L445I) were created by assembling the fragments containing the mutations which were obtained using PCR to amplify the corresponding fragments from the plasmids pSM3 and pGK8. Upstream and downstream fragments containing desired mutant combinations were combined with a pTrc99A backbone fragment to create full length plasmids using Gibson Assembly (New England Biolabs). In addition, standard QuickChange site-directed mutagenesis protocol was used to introduce L445I, A18T, and V275F mutations to plasmids pSM3 (pGlf_A165M_) and pGlf_wt_, generating pGlf_A165M L445I_, pGlf_A18T_, and pGlf_V275F_. The primers used for plasmid construction are listed in Table S1. Mutations in each plasmid were confirmed by Sanger sequencing at the ASU Genomics Core.

### Fermentation

Cells were incubated for ∼18 h under microaerobic conditions on LB agar plates in a container filled with argon gas. Then a few colonies were transferred into 100 ml LB supplemented with 100 mM MOPS in 250 ml flasks without including sugars to avoid unexpected accumulation of beneficial mutations to alter glucose or xylose catabolism. These seed cultures were incubated for 12–18 h before being transferred into 300 ml AM1 media in 500 ml fermentation vessels for single sugar fermentation (Martinez et al., 2007) or modified AM1 media [twofold (NH_4_)_2_HPO_4_ and NH_4_H_2_PO_4_ relative to the original recipe] for glucose–xylose cofermentation (Martinez et al., 2019). When performing cosugar fermentations, 66 g l^–1^ glucose and 34 g l^–1^ xylose were used, a typical mass ratio representative of lignocellulosic sugar compositions (Saha, 2003). 100 mg l^–1^ampicillin and 10 µM IPTG were included in all fermentation tests for the cells with plasmids to maintain the plasmids and induce suitable expression for transporter genes as previously described (Kurgan, et al., 2019a,b). All batch fermentations were inoculated using an initial OD_550nm_ of 0.05 and fermentation was controlled at 37°C and pH 7.0 with automatic additions of 6 M potassium hydroxide.

### Homology Modeling and Structural Analysis

Homology models of Glf were constructed using SWISS-MODEL (Biasini et al., 2014) with the crystal structures of XylE bound to glucose (PDB ID: 4GBZ) or xylose (PDB ID: 4GBY) as template. Structures were viewed and edited using the Chimera software suite (Pettersen et al., 2004). The location of predicted structural features was overlaid onto a Clustal Omega alignment of Glf and XylE. Visualizations of the alignment similarity and identity were generated using the Multiple Alignment Show tool from the Sequence Manipulation Suite (http://www.bioinformatics.org/sms2/).

### Analysis

Quantification of sugars was performed using HPLC (Thermo Fisher Scientific UltiMate 3000) equipped with an Aminex HPX-87H column (Bio-Rad). 4.0 mM sulfuric acid was used as mobile phase at a flow rate of 0.4 ml min^–1^. Optical density was quantified using a UV − Vis spectrophotometer (Beckman Coulter DU-730). All experiments were performed in at least three biological replicates, and the average and standard deviation are shown in figures.

## Results and Discussion

### Construction of a Platform Strain to Select for Glucose-Resistant Glf Variants

**Fig. 1:**
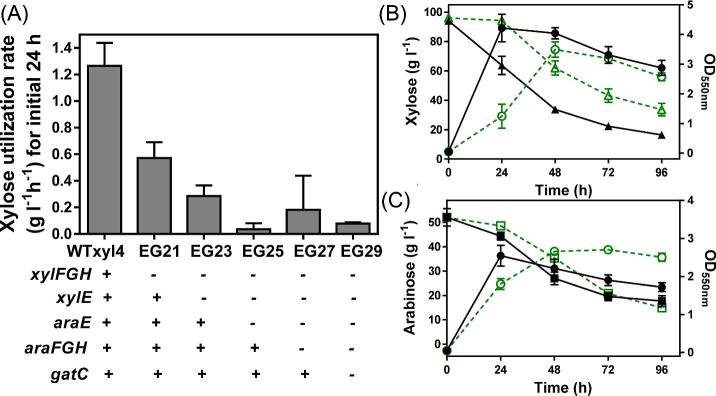
Construction of a screening platform strain deficient in both glucose and xylose uptake. (A) Xylose consumption rates for the initial 24 hours of WTxyl4 and its derivatives engineered by consecutive inactivation of reported and putative xylose transporters. Fermentation of WTxyl4 (solid lines with filled symbols) and EG29 (dotted lines with open symbols) using mineral salts media supplemented with (B) 100 g l^−1^ xylose or (C) 50 g l^−1^ arabinose. Symbols: OD550_nm_ (circle), xylose (triangle), arabinose (square).

To create a bacterial strain to facilitate rapid identification of glucose-resistant Glf variants, we sought to create a glucose and xylose transport deficient strain which relies on foreign sugar transport systems for cell growth, as has been done previously in yeast (Farwick et al., 2014). We began with WTxyl4, a previously engineered *E. coli* strain created in our lab (Flores et al., 2019) with main glucose uptake systems inactivated (ATCC 9637 Δ*ptsI* Δ*ptsG* Δ*galP*). In addition, in this strain wild-type *xylR* was replaced with a mutant copy xylR* (*xylR*::xylR*) which relieves CCR and activates xylose catabolic genes such as *xylA* and *xylB* even in the presence of glucose (Flores et al., 2019; Sievert et al., 2017). Approximately 90% glucose provided remained unused over 96 h fermentation (Flores et al., 2019), confirming that PTS and GalP (galactose-proton symporter) are the main glucose uptake mechanisms in *E. coli* W (ATCC 9637). We further modified this strain by inactivating all reported *E. coli* xylose transporters. Inactivation of *xylFGH* decreased the initial xylose utilization rate (0–24 h) by 50% (Fig. 1A). This rate was decreased to approximately 25% of the rate of WTxyl4 by further deletion of *xylE* (Fig. 1A). AraE was reported to have activities toward xylose (Hasona et al., 2004), and further deletion of *araE* in this background decreased initial xylose utilization rate down to 8% (Fig. 1A).

**Fig. 2:**
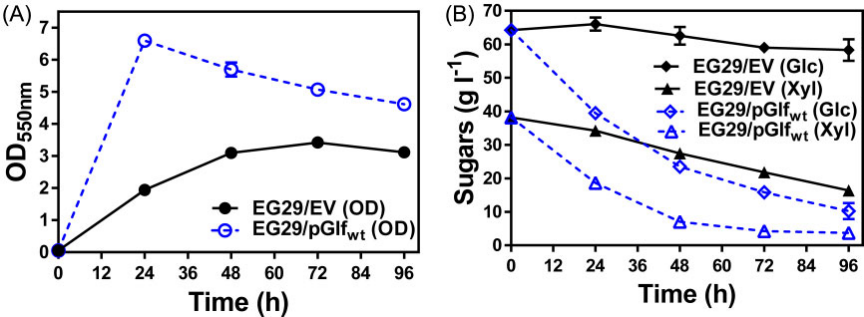
Glf enables glucose and xylose coutilization in EG29. Fermentation of EG29 transformed with empty vector (EV) pTrc99A (solid lines with filled symbols) or pGlf_wt_ (dotted lines with open symbols) using mineral salts media containing sugar mixtures with 66 g l^−1^ glucose and 34 g l^−1^ xylose. (A) OD_550nm_ and (B) sugar concentrations in broth were measured at indicated time intervals. 100 mg l^−1^ampicillin and 10 µM IPTG were included in all fermentation tests to maintain the plasmids and induce *glf* expression. Symbols: OD_550nm_ (circle), xylose (triangle), glucose (diamond).

To completely abolish xylose uptake in this background, we also deleted *araFGH* and *gatC* which encode putative xylose transporters (Utrilla et al., 2012), which resulted in the strain EG29. Surprisingly, xylose (Fig. 1B) or arabinose utilization (Fig. 1C) of EG29 was only disrupted in the first 24 h, and significant xylose and arabinose fermentation and cell growth were observed after 24 h, suggesting the presence of alternative and yet unknown transporters responsible for transporting xylose and arabinose. A plasmid encoding Glf enabled EG29 to use 90% of 100 g l^–1^ total sugars when fermenting glucose–xylose mixtures (2:1 by mass), thus leading to much improved cell growth compared to empty vector control (Fig. 2A and B). This result indicates that Glf is able to serve as an efficient transporter for both glucose and xylose. Our previous work suggests that utilization of GalP as a substitute glucose transporter is undesirable for conversion of lignocellulosic sugar mixtures because GalP represses xylose fermentation and uses proton gradients (Kurgan, Sievert, et al., 2019). Thus, Glf is a more suitable substitute glucose transporter for lignocellulosic bioconversion compared to GalP since Glf is more energy efficient and has no repressive effect on xylose fermentation. In contrast, Glf itself can function as an efficient xylose transporter (Fig. 2B).

**Fig. 3:**
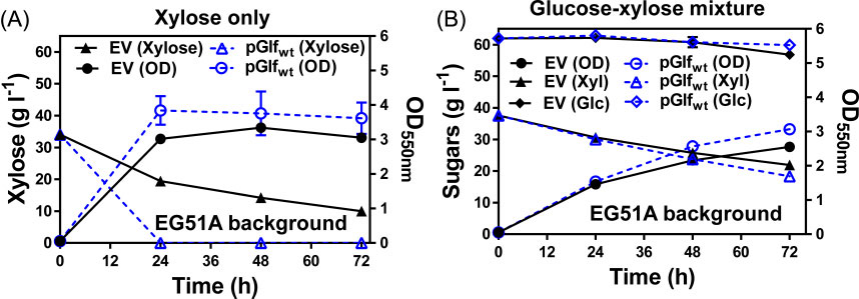
Xylose transport of Glf is inhibited by glucose. Fermentation of EG51A (EG29 *glk*^−^) transformed with empty vector (EV) pTrc99A (solid lines with filled symbols) or pGlf_wt_ (dotted lines with open symbols) using mineral salts media containing (A) 34 g l^−1^ xylose only or (B) sugar mixtures with 66 g l^−1^ glucose and 34 g l^−1^ xylose. OD_550nm_ and sugar concentrations in broth were measured at indicated time intervals. 100 mg l^−1^ampicillin and 10 µM IPTG were included in all fermentation tests to maintain the plasmids and induce *glf* expression. Symbols: OD_550nm_ (circle), xylose (triangle), glucose (diamond).

To facilitate a genetic screen for resistance to glucose inhibition, high extracellular glucose concentrations were maintained by completely disrupting glucose degradation in EG29 through deletion of glucose kinase gene, resulting in the strain EG51A. When fermenting xylose alone, EG51A transformed with pGlf_wt_ showed much improved xylose utilization compared to EG51A with empty vector pTrc99A (Fig. 3A). However, xylose utilization assisted by Glf is almost completely disrupted in the presence of glucose, and thus cells used a similar amount of xylose as those carrying an empty vector (Fig. 3B). Therefore, the transport function of Glf is severely inhibited by glucose in EG51A, allowing for its use as a growth-based selection platform to identify Glf variants that are resistant to glucose inhibition.

### Directed Evolution of Glf to Overcome Glucose Inhibition

**Fig. 4:**
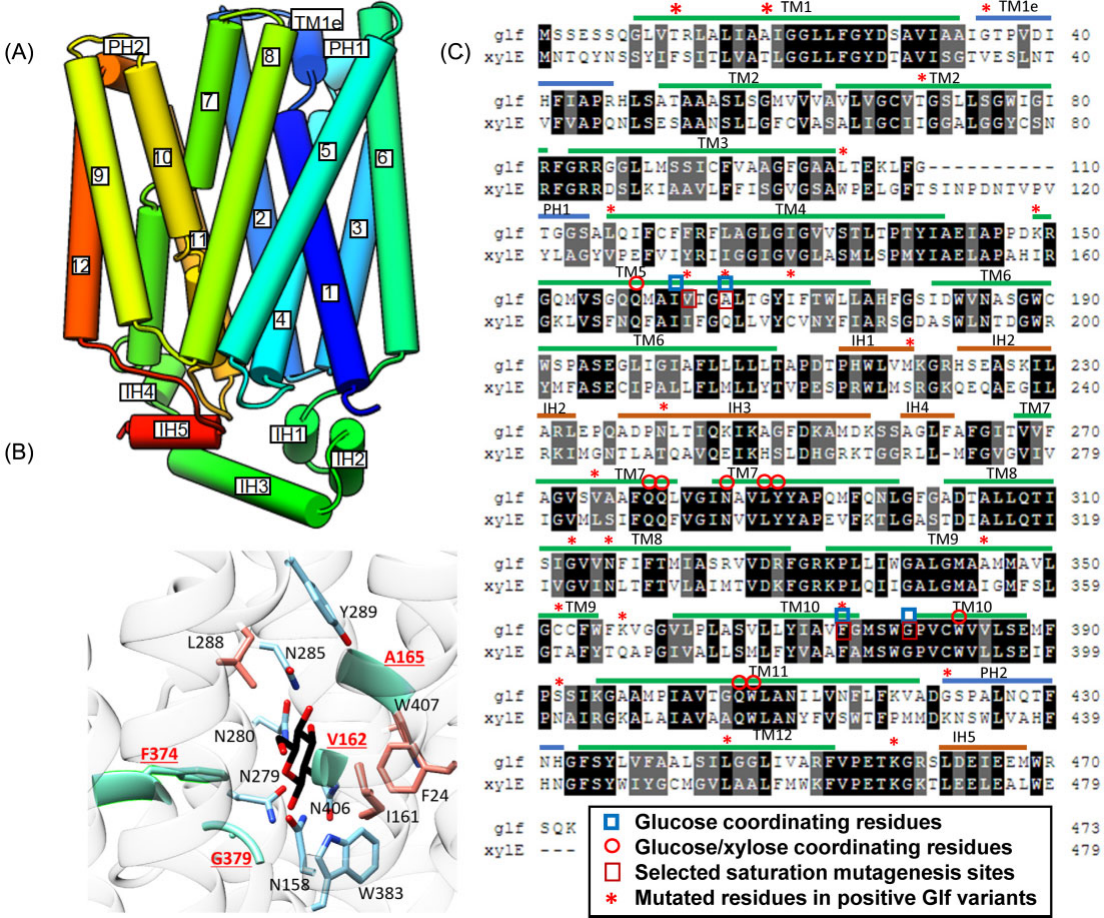
Structural information for Glf mutagenesis. (A) A homology model of Glf depicts a similar structural organization to XylE consisting of 12 transmembrane domains (TM), 2 periplasmic helices (PH), and 5 intracellular helices (IH). (B) Putative glucose coordination of Glf. Contacts forming hydrogen bonds (residues in sky blue) or hydrophobic contacts (residues in salmon) with D-glucose (black) according to the XylE crystal structure (PDB ID: 4GBZ) are displayed in the image. All glucose coordinating sites except A165 are conserved in the D-glucose-binding pocket of XylE. Residues V162, A165, F374, and G379 (in aquamarine) were selected for site saturation mutagenesis to potentially disrupt glucose coordination. (C) Protein sequence alignment of Glf and XylE using Clustal Omega, with the mutations found in positive Glf variants, residues involved in glucose and/or xylose coordination, and sites selected for saturation mutagenesis being distinctly labeled.

To create Glf variants, we took a two-pronged approach employing both random and site-saturation mutagenesis. For random mutagenesis, approximately 12 000 variants were created using error-prone PCR (GK plasmid library). For site-saturation mutagenesis, homology models of Glf constructed using the XylE crystal structures (PDB IDs: 4GBY and 4GBZ) were employed to select target sites since the structure of Glf is uncharacterized. XylE, a low affinity xylose-proton symporter, shares high sequence identify (43%) with Glf and also belongs to the MFS family. The model structure of Glf consists of twelve TMs, two extracellular helices (EC), and five intracellular helices (IH) in addition to one kinked region of TM1 that may reside on the extracellular side (TM1e) (Fig. 4A). Previous work has elucidated the structures of XylE bound to xylose and glucose at a high resolution (Sun et al., 2012). Residues involved in coordination of the 6-hydroxymethyl group of glucose that are not involved with xylose coordination were found to reside within TM5, TM8, and TM10 (Sun et al., 2012). Similar to XylE, most putative sugar coordinating residues are located in the C-terminal region of the Glf model structure (Fig. 4B). We targeted the sites that are involved in the coordination of glucose but not xylose in the Glf homology model structure. Guided by structural models and a sequence alignment generated using Clustal Omega algorithm (Sievers et al., 2011), we selected residues A165, F374, and G379 (Q175, F383, and G388 in XylE) that correspond to glucose coordination (Fig. 4B). We also chose V162 in Glf, located between the glucose-binding residues I171 and Q175 in XylE, due to the finding that mutation of this corresponding residue was capable of releasing glucose repression in multiple yeast uniporters (Farwick et al., 2014; Li et al., 2016). For each site, the variant sublibrary (later four sublibraries combined to form SM plasmid library) contained at least 200 clones to ensure sufficient coverage. In addition, we also constructed and individually tested two variants that had been previously identified to enhance pentose utilization, A18T and V275F (Dunn & Rao, 2015).

**Fig. 5:**
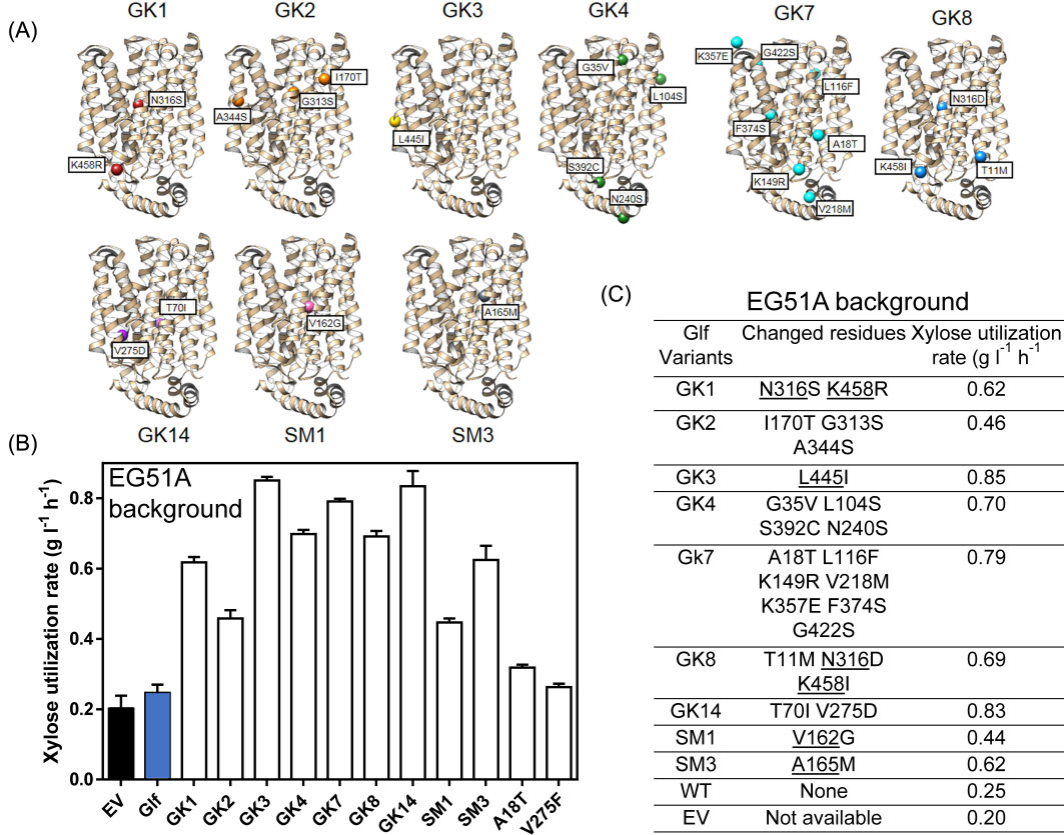
Directed evolution yielded Glf variants resistant to glucose inhibition. (A) Positive Glf variants isolated after fermentative growth selection using glucose–xylose mixtures. (B) Xylose utilization rates (initial 24 h) of EG51A transformed with Glf variants as well as control plasmids including empty vector (EV) using mineral salts media containing sugar mixtures with 66 g l^−1^ glucose and 34 g l^−1^ xylose. 100 mg l^−1^ampicillin and 10 µM IPTG were included in all fermentation tests to maintain the plasmids and induce *glf* expression. (C) Summary of Glf variants and EV for their xylose utilization rates under glucose inhibition in EG51A background. The residues potentially important for glucose inhibition are underscored.

After growth selection to enrich Glf variants resistant to glucose inhibition under fermentation conditions using glucose–xylose mixtures as described in Materials and Methods, nine distinct variants (GK1, GK2, GK3, GK4, GK7, GK8, and GK14 from GK library; SM1 and SM3 from SM library) were identified to overcome glucose inhibition (Figs. 4C and 5A). EG51A transformed with plasmids containing these nine variants had increased initial xylose utilization rates ranging from 1.8-fold to 3.4-fold compared to cells with wild-type Glf in the presence of glucose (Fig. 5B and C). If subtracting the xylose utilization contributed from the background (0.20 g l^–1^ h^–1^ EG51A with EV), the gained xylose utilization rates of these nine variants were increased ranging from 4.8-fold to 13-fold relative to wild-type Glf (Fig. 5C). Variants A18T and V275F did not significantly change the glucose inhibition phenotype, and thus were not analyzed further (Fig. 5B). For mutants derived from site-saturation mutagenesis, variants V162G (SM1) and A165M (SM3) showed 4.8- and 8.4-fold increase in xylose utilization rate relative to wild type, respectively. Among identified variants created by random mutagenesis, GK3, GK7, and GK14 showed most significant resistance to glucose inhibition with xylose utilization rates increased to 13.0-, 11.8-, and 12.6-fold relative to that of wild type (Fig. 5B and C).

### Diverse Glucose-Resistant Glf Variants Suggest Multiple Mechanisms to Overcome Glucose Inhibition

Putative mutations from nine positive Glf variants conferring resistance to glucose inhibition are widely distributed in most TMs, IHs, and PH2 (Fig. 4C). Except for GK3, all GK variants have multiple mutations, which complicates the analysis of the important residues involved in glucose inhibition. Overall, mutations in TM5, TM8, and TM10 (TMs known to coordinate glucose in XylE structure) account for 67% of variants, suggesting that this region is important for glucose inhibition. Previous work has extensively studied the mutations releasing glucose inhibition in fungal sugar transporters (Gardonyi et al., 2003; Runquist et al., 2010). Many of the residues important for glucose inhibition in these eukaryotic sugar uniporters were found to correspond to identified mutations in this work, suggesting a conserved mechanisms of glucose inhibition. For instance, in fungal MFS transporters Gxs1 (from *Candida intermedia)*, Gal2, Hxt7, Hxt5, Hxt11, and Hxt3, mutation of the conserved asparagine in TM8 (corresponding to N316 in Glf) has been observed to release glucose inhibition with varying degrees of success (Farwick et al., 2014; Li et al., 2016; Nijland et al., 2016; Shin et al., 2015). Both GK1 and GK8 have N316 mutated (N316S and N316D, respectively), suggesting a convergent mechanism of GK1 and GK8 to release glucose inhibition. It is likely that these changes alter the structure of the sugar coordinating pocket, especially near the 6-hydroxymethyl group of D-glucose. GK1 and GK8 also have K458 mutated (K458R and K458I, respectively). This C-terminal lysine residue is located between TM12 and IH5, and is predicted to position directly below the extending cytoplasmic junction between TM8 and TM9, which may indicate an interaction between this residue and residues in TM8-9. In other GK variants, GK7 has serine substitution at F374 that is involved in glucose coordination and is structurally conserved in Glf (Fig. 4B). However, site-saturation mutagenesis at F374 did not yield positive variants, suggesting other mutations in GK7 may be needed to achieve the resistance phenotype. GK2 has a mutation in TM8 (G313S) that is homologous to a described mutation in Glut1 (G314S) known to cause decreased glucose transport without affecting cation permeability (Weber et al., 2008) (Fig. S1). Other mutations in GK variants such as L445I (GK3) do not appear to have a direct role in glucose coordination. The mechanism to resist glucose inhibition for these mutations is unclear.

Discovery of SM1 and SM3 variants using site-saturation mutagenesis for V162 and A165 sites confirmed our hypothesis: these residues in Glf are involved in glucose coordination and amino acid substitutions at these sites weaken glucose inhibition. Mutations similar to V162G in the Gxs1, Gal2, Hxt7 and Hxt5 result in resistance to glucose inhibition (Farwick et al., 2014; Li et al., 2016). Although the alanine at the position 165 in Glf cannot form a hydrogen bond with 6-hydroxymethyl group of glucose such as the glutamine at the position 175 in XylE, it is still important for glucose coordination. Based on Glf homology model, A165 extends directly into the sugar coordinating pocket near the 6-hydroxymethyl group of glucose. Methionine substitution may potentially hinder glucose from entering the binding pocket due to its larger-side chain. V162G may have a similar working mechanism that influence glucose binding by altering size or structure of the translocation pocket.

Thus, diverse mutations have been identified to influence glucose inhibition through (1) altering the residues in TM5, TM8, and TM10 that are involved glucose coordination (likely GK1, GK2, GK7, GK8, SM1, and SM3) and (2) uncharacterized mechanisms (GK3, GK4, and Gk14).

### Investigation of Combinatory Mutations in Glf to Overcome Glucose Inhibition

**Fig. 6:**
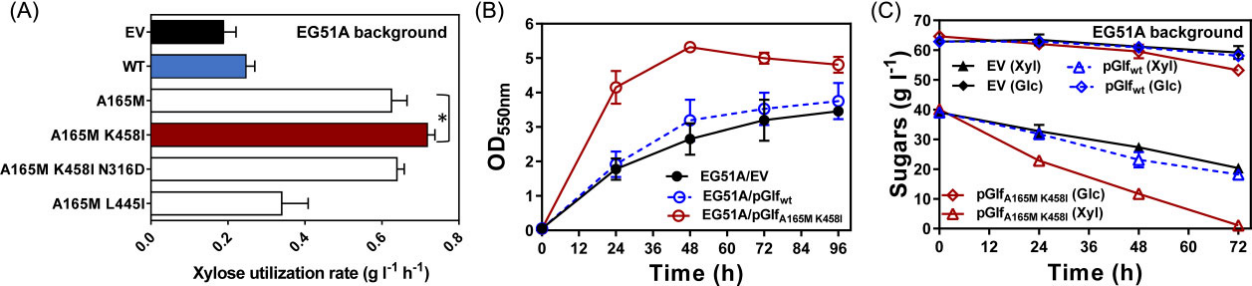
The effect of combinatory mutations on glucose inhibition. (A) Xylose utilization rates (initial 24 h) of EG51A transformed with Glf variants as well as control plasmids including empty vector (EV) using mineral salts media containing sugar mixtures with 66 g l^−1^ glucose and 34 g l^−1^ xylose. *p < .05 as estimated by one-tailed Student's t test. Batch fermentations of EG51A transformed with EV or plasmids encoding wild-type Glf or A165M K458I mutant. 100 mg l^−1^ ampicillin and 10 µM IPTG were included in all fermentation tests to maintain the plasmids and induce *glf* expression. Cells were fermented using mineral salts medium with 66 g l^−1^ glucose and 34 g l^−1^ xylose. (B) OD_550nm_ and (C) sugar concentrations in broth were measured at indicated time intervals. Symbols: OD_550nm_ (circle), xylose (triangle), glucose (diamond).

A165M, N316D, K458I, and L445I were next selected for further test of the combinatory effect on resistance of glucose inhibition. A165M (from SM3) and L445I (from GK3) were chosen because they are single mutations that cause significant resistance to glucose inhibition. N316D and K458I were chosen because these two mutated sites were found in both GK1 and GK8 (Fig. 5C), suggesting that these changes are causative to the resistance phenotype. The xylose utilization rate of strain EG51A transformed with a plasmid combining the A165M and K458I mutations (pGlf_A165M K458I_) was 15% higher than EG51A transformed with a plasmid containing the A165M Glf alone when cells fermented glucose–xylose mixtures (Fig. 6A). Compared to wild-type Glf, the gained xylose utilization rate of A165M K458I Glf variant was increased 11.5-fold if subtracting background xylose utilization. Cell growth was also dramatically increased due to increased sugar catabolism (Fig. 6B) and all xylose was used within 72 h (Fig. 6C). This result suggests that a positive combinatory effect to A165M can be achieved by combining with other positive mutations. However, when N316D was further combined with A165M and K458I, the xylose utilization rate decreased to the level of A165M (Fig. 6A). An interesting detrimental effect was observed when A165M and L445I were combined. The EG51A strain transformed with double-mutant plasmid pGlf_A165M L445I_ had even a lower xylose utilization rate than cells with SM3 (A165M) or GK3 (L445I) single mutations (Figs. 5B and 6A). This result suggests that negative epistatic interactions may occur for positive mutations due to their distinct working mechanisms and the dynamic nature of proteins.

Next, we investigated if the glucose transport by Glf was disrupted when introducing these mutations to overcome glucose inhibition. All combinatory mutations as well as isolated GK and SM Glf variants were transformed into EG29 and their glucose utilization abilities were evaluated under fermentation conditions using glucose–xylose mixtures. Interestingly, most Glf variants, except for GK2, GK14, and A165M N316D K458I, retain similar glucose utilization rates compared to wild-type Glf (Fig. S1). This finding further provides evidence that the mechanism of glucose inhibition for MFS proteins seems to be independent of glucose transport function. This is corroborated by findings by Young et al. that eliminating glucose transport but enabling xylose transport does not simply release glucose inhibition in Gxs1 (Young et al., 2014). This is also observed in XylE, which is bound and inhibited by glucose even though it does not actively transport glucose (Sun et al., 2012). The GK2, GK14, and A165M N316D K458I variants had decreased glucose utilization rates by 30–40%, suggesting that the mutations in these Glf variants may be involved in both glucose transport and glucose inhibition. Xylose uptake abilities of these combinatory mutations in EG29 are similar to wild-type Glf (data not shown). Comprehensive evaluation of these glucose-resistant Glf variants in *E. coli* production strains with varied lignocellulosic sugar mixtures will be the next step for future study. In addition, since typical lignocellulosic hydrolysates generated by thermochemical processes contain toxic side products (Nieves et al., 2015) which may influence transporter functions, the performance of these Glf variants using lignocellulosic hydrolysates instead of model sugar mixtures as fermentation substrates needs to be further investigated.

In conclusion, nine Glf variants that are at least partially resistant to glucose inhibition were identified through directed evolution using a CCR-free *E. coli* platform strain with deficiency in glucose and xylose transport. Most mutations likely alter the sugar translocation pocket that is responsible for coordinating the 6-hydroxymethyl group of D-glucose although other more complex mechanisms may also be present. These Glf variants are potentially useful to help conserve cellular energy and overcome glucose inhibition during lignocellulose bioconversion.

## Funding

This work was supported by the National Science Foundation (MCB-1942825) and the start-up fund from Arizona State University.

## Conflict of Interest

The authors declare no conflict of interest.

## Supplementary Material

kuab066_Supplemental_File
