# Supplementary material for: Directed evolution of Zymomonas mobilis sugar facilitator Glf to overcome glucose inhibition
Source: J Ind Microbiol Biotechnol. 2021 Sep 16;49(2):kuab066. doi: 10.1093/jimb/kuab066 (PMC9118996; doi:10.1093/jimb/kuab066)
Supplement: kuab066_Supplemental_File [file kuab066_Supplemental_File.docx]

Table S1. Primers used in this study

| **Primer** | **Sequence (5' to 3')** |
| --- | --- |
| *xylFGH* deletion | GCTGGTCAAAATAGGCTTGC (forward primer outside of ORF for colony PCR confirmation) |
|  | GACCATAAAAAGCAAAGCGG (reverse primer outside of ORF for colony PCR confirmation) |
|  | GTGATTGTTACTTATTAAAGCTGTCCTCTAACTACAGAAGGCCCTACACCGTGTAGGCTGGAGCTGCTTC (primer to amplify FRT-kan-FRT from pKD4) |
|  | AACTCAAAACCGGTAATACGTAACCGGCTTTGAGAAAATTTTTATCAAAACATATGAATATCCTCCTTAGT (primer to amplify FRT-kan-FRT from pKD4) |
| *xylE* deletion | TCACCATCGTCTTCTTGCTG |
|  | ACTCAATCTTGGCCTGCTGT |
|  | CCAACATCAATGCACTGATAAAAGATCAGAATGGTCTAAGGCAGGTCTGAGTGTAGGCTGGAGCTGCTTC |
|  | GGAGTAAAAAGACAGCACGCCGAACTGGCGCGGCGTGCTGGACAGGAAGACATATGAATATCCTCCTTAGT |
| *araE* deletion | CCTCCAGCGCTTCAGATTAT |
|  | AGAAAGGCAGAGTGCAACGT |
|  | ATTGTTCACGTATTTTTTCACTATGTCTTACTCTCTGCTGGCAGGAAAAAGTGTAGGCTGGAGCTGCTTC |
|  | CTCTATTAACGAAAAAAGGGCCGGATGTACAGCACATCCGGCCCGTGAAACATATGAATATCCTCCTTAGT |
| *araFGH* deletion | AATGACGTGCATTTTCCACA |
|  | GAAGCGATAAGGTTTTGCCA |
|  | TGTCATTCGTTTTTGCCCTACACAAAACGACACTAAAGCTGGAGAGAACCGTGTAGGCTGGAGCTGCTTC |
|  | GGCGGCTGGCTGTGGTGGGAAAAAACGTTAAATTGTTGTGGAAAAAAGCACATATGAATATCCTCCTTAGT |
| *gatC* deletion | CCCGATATCGTCGAGACACT |
|  | CAATAAAAGCCCCATCCTCA |
|  | GTATCGAAGCATTACAAAATAAAATTCTGACTATCTTACAGGGGTGACCTGTGTAGGCTGGAGCTGCTTC |
|  | CATAAAAACTCCTGATTGTTAAGGGGGATAACCTCCCCCTCAGGTAAAAACATATGAATATCCTCCTTAGT |
| *glk* deletion | ACCGATGATCCAGAGAGGTG |
|  | TAATTGCGTCCTGCAAAGTG |
|  | ATTTACAGTGTGAGAAAGAATTATTTTGACTTTAGCGGAGCAGTTGAAGAGTGTAGGCTGGAGCTGCTTC |
|  | TGATTTAAAAGATTATCGGGAGAGTTACCTCCCGATATAACAGGAAGGATCATATGAATATCCTCCTTAGT |
| pGlf_A165M K458I_ and pGlf _A165M K458I N316D_ construction* | (SH01 primer) TCAGAAGGCCTGATCGGTAT |
|  | (SH02 primer) ATACCGATCAGGCCTTCTGA |
|  | (SH03 primer) TGCTTATTTGGGGTGCTCTC |
|  | (SH04 primer) GAGAGCACCCCAAATAAGCA |
|  | (SH05 primer) GGATCCTCTAGAGTCGACCTGCAGG |
|  | (SH06 primer) CCTGCAGGTCGACTCTAGAGGATCCCTACTTCTGGGAGCGCCACATC |
| *glf* cloning into pTrc99A to create pGlf_wt_ | GGATCCTCTAGAGTCGACCTGCAGG |
|  | CGGGTACCGAGCTCGAATTCCATG |
|  | CATGGAATTCGAGCTCGGTACCCGGCCATGAGTTCTGAAAGTAGTCAGGG |
|  | CCTGCAGGTCGACTCTAGAGGATCCCTACTTCTGGGAGCGCCACATC |
| A18T mutation | CGCGACTAGCCCTAATCGCTACCATAGGCGGCTTGCTTTTCGGT |
|  | ACCGAAAAGCAAGCCGCCTATGGTAGCGATTAGGGCTAGTCGCG |
| V275F mutation | CCGTTGTTTTTGCCGGTGTATCCTTTGCTGCCTTCCAGCAGTTAGTCG |
|  | CGACTAACTGCTGGAAGGCAGCAAAGGATACACCGGCAAAAACAACGG |
| L445I mutation | GTTTTCGCAGCATTAAGTATCATAGGTGGCTTGATTGTTG |
|  | CAACAATCAAGCCACCTATGATACTTAATGCTGCGAAAAC |
| V162NNK mutations | CAGCAGATGGCCATTNNKACGGGTGCTTTAACC |
|  | GGTTAAAGCACCCGTMNNAATGGCCATCTGCTG |
| A165NNK mutations | GCCATTGTGACGGGTNNKTTAACCGGTTATATC |
|  | GATATAACCGGTTAAMNNACCCGTCACAATGGC |
| F374NNK mutations | CTTTATATTGCAGTCNNKGGTATGTCATGGGGC |
|  | GCCCCATGACATACCMNNGACTGCAATATAAAG |
| G379NNK mutations | TTTGGTATGTCATGGNNKCCTGTCTGCTGGGTT |
|  | AACCCAGCAGACAGGMNNCCATGACATACCAAA |

*SH03 and SH06 were used to amplify the fragment from GK8, and SH04 and SH05 were used to amplify the fragment from SM3. Then both fragments were assembled using Gibson Assembly kit. Similarly, SH01 and SH06 were used to amplify the fragment from GK8, and SH02 and SH05 were used to amplify the fragment from SM3.

Fig. S1


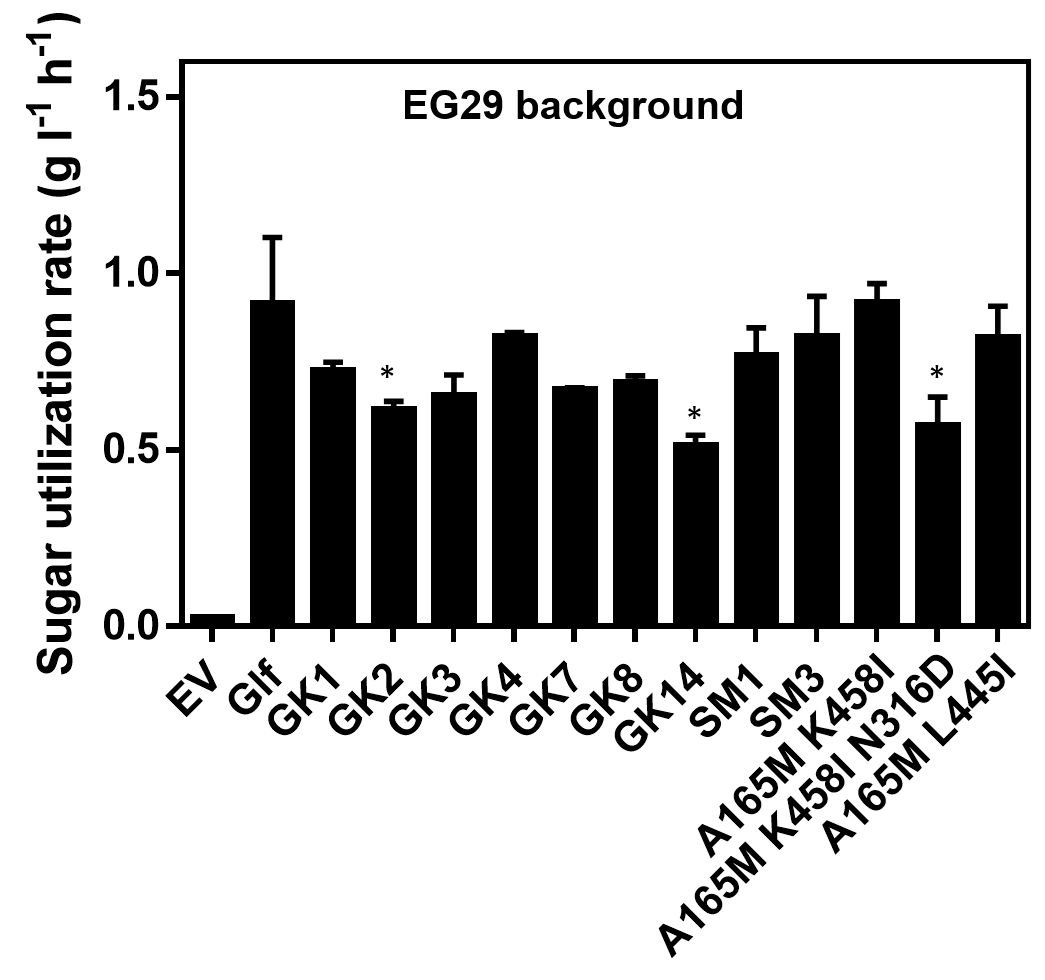


Figure S1. Glucose fermentation of EG29 expressing Glf variants. Glucose utilization rates (initial 24 h) of EG29 transformed with Glf variants as well as control plasmids including empty vector (EV) using mineral salts media containing sugar mixtures with 66 g l^-1^ glucose and 34 g l^-1^ xylose. *p < 0.05 as estimated by one-tailed Student’s t test.
